# Correction: Cost-utility analysis of proton beam therapy for locally advanced esophageal cancer in Japan

**DOI:** 10.1371/journal.pone.0330124

**Published:** 2025-08-11

**Authors:** Takuya Sawada, Masahide Kondo, Masaaki Goto, Motohiro Murakami, Toshiki Ishida, Yuichi Hiroshima, Shu-Ling Hoshi, Reiko Okubo, Toshiyuki Okumura, Hideyuki Sakurai

In Fig 2, the figure legend is incorrect. The labels for the black square ‘Lower’ and the white square ‘Upper’ are swapped. Please see the correct Fig 2 here.

**Fig 2:**
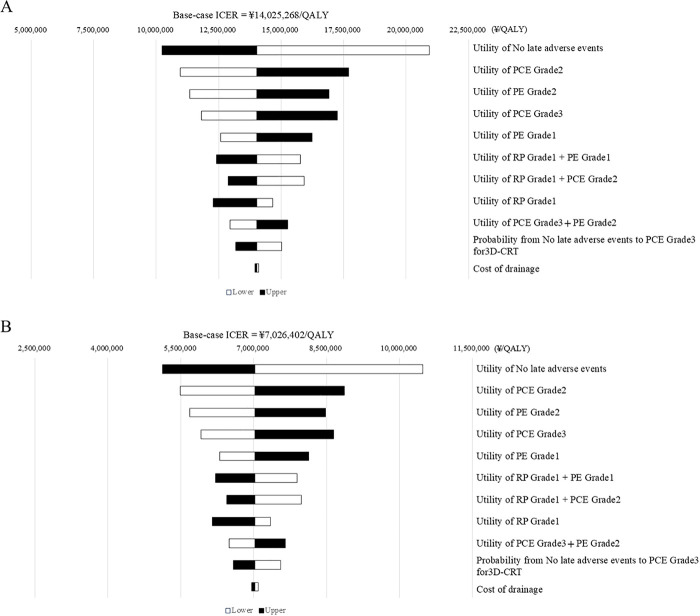
Results of one-way sensitivity analysis. (A) Rare cancer base-case. (B) Non-rare cancer base-case. Abbreviations: PCE, pericardial effusion; PE, prepupal effusion; 3D-CRT, three-dimensional conformal radiotherapy. Ten variables with large incremental cost-effectiveness ratio (ICER) changes from the base case values are shown. The ICERs were all > \7,500,000/QALY in the rare cancer base-case, and eight items in the non-rare cancer base-case were > \7,500,000/QALY.
