# Supplementary material for: Cost-utility analysis of proton beam therapy for locally advanced esophageal cancer in Japan
Source: PLoS One. 2024 Sep 27;19(9):e0308961. doi: 10.1371/journal.pone.0308961 (PMC11433116; doi:10.1371/journal.pone.0308961)
Supplement: Attachment — Submitted filename: Response to Reviewers.docx [file pone.0308961.s001.docx]

Dear Dr. Sina Azadnajafabad

We would like to thank you profusely for reviewing our research paper. Regarding the comments received from the editors and reviewers, we would like to respond to you as follows. We would appreciate it if you would take the time to review it again.

Kind regards,

Masahide Kondo

Journal Requirements:

1. When submitting your revision, we need you to address these additional requirements.

Please ensure that your manuscript meets PLOS ONE's style requirements, including those for file naming. The PLOS ONE style templates can be found at 
<https://journals.plos.org/plosone/s/file?id=wjVg/PLOSOne_formatting_sample_main_body.pdf> and 
<https://journals.plos.org/plosone/s/file?id=ba62/PLOSOne_formatting_sample_title_authors_affiliations.pdf>

2. Please note that PLOS ONE has specific guidelines on code sharing for submissions in which author-generated code underpins the findings in the manuscript. In these cases, all author-generated code must be made available without restrictions upon publication of the work. Please review our guidelines at <https://journals.plos.org/plosone/s/materials-and-software-sharing#loc-sharing-code> and ensure that your code is shared in a way that follows best practice and facilitates reproducibility and reuse.

3. When completing the data availability statement of the submission form, you indicated that you will make your data available on acceptance. We strongly recommend all authors decide on a data sharing plan before acceptance, as the process can be lengthy and hold up publication timelines. Please note that, though access restrictions are acceptable now, your entire data will need to be made freely accessible if your manuscript is accepted for publication. This policy applies to all data except where public deposition would breach compliance with the protocol approved by your research ethics board. If you are unable to adhere to our open data policy, please kindly revise your statement to explain your reasoning and we will seek the editor's input on an exemption. Please be assured that, once you have provided your new statement, the assessment of your exemption will not hold up the peer review process.

4. PLOS requires an ORCID iD for the corresponding author in Editorial Manager on papers submitted after December 6th, 2016. Please ensure that you have an ORCID iD and that it is validated in Editorial Manager. To do this, go to ‘Update my Information’ (in the upper left-hand corner of the main menu), and click on the Fetch/Validate link next to the ORCID field. This will take you to the ORCID site and allow you to create a new iD or authenticate a pre-existing iD in Editorial Manager. Please see the following video for instructions on linking an ORCID iD to your Editorial Manager account: <https://www.youtube.com/watch?v=_xcclfuvtxQ>

Regarding 1.~4.,

We have revised the font size, abbreviations, and other formatting based on the URLs you provided. In addition, we have included the URLs that can be referred so as to make them accessible for all references. Finally, we will update ORCID as soon as possible after submitting this revise.

[Note: HTML markup is below. Please do not edit.]

Reviewers' comments:

Reviewer's Responses to Questions

**Comments to the Author**

1. Is the manuscript technically sound, and do the data support the conclusions?

The manuscript must describe a technically sound piece of scientific research with data that supports the conclusions. Experiments must have been conducted rigorously, with appropriate controls, replication, and sample sizes. The conclusions must be drawn appropriately based on the data presented.

Reviewer #1: Partly

Reviewer #2: Yes

Reviewer #3: No

2. Has the statistical analysis been performed appropriately and rigorously?

Reviewer #1: Yes

Reviewer #2: Yes

Reviewer #3: Yes

3. Have the authors made all data underlying the findings in their manuscript fully available?

The [PLOS Data policy](http://www.plosone.org/static/policies.action#sharing) requires authors to make all data underlying the findings described in their manuscript fully available without restriction, with rare exception (please refer to the Data Availability Statement in the manuscript PDF file). The data should be provided as part of the manuscript or its supporting information, or deposited to a public repository. For example, in addition to summary statistics, the data points behind means, medians and variance measures should be available. If there are restrictions on publicly sharing data—e.g. participant privacy or use of data from a third party—those must be specified.

Reviewer #1: Yes

Reviewer #2: Yes

Reviewer #3: Yes

4. Is the manuscript presented in an intelligible fashion and written in standard English?

PLOS ONE does not copyedit accepted manuscripts, so the language in submitted articles must be clear, correct, and unambiguous. Any typographical or grammatical errors should be corrected at revision, so please note any specific errors here.

Reviewer #1: Yes

Reviewer #2: Yes

Reviewer #3: Yes

5. Review Comments to the Author

Please use the space provided to explain your answers to the questions above. You may also include additional comments for the author, including concerns about dual publication, research ethics, or publication ethics. (Please upload your review as an attachment if it exceeds 20,000 characters)

**Reviewer #1**: #1 “In Japan, 3D CRT is the standard radiotherapy method with strong evidence for definitive CRT for LAEC [3, 4].” & “In addition, the Japanese guidelines for the treatment of esophageal cancer recommend PBT over IMRT as the treatment of choice for patients with poor cardiopulmonary function [9]” while ref-3 & ref-4 & ref-9 were papers from Japan.” & “We compared PBT with the standard treatment, 3D-CRT”
>> Please note that another guideline from Japan published in 2022 (ref-1 of the current manuscript) stated “Recently, high-precision radiotherapy, such as intensity-modulated radiotherapy with X-rays and particle therapy with proton or heavy ion beams, has been reported to reduce these adverse effects” without clear statement that that PBT was superior to IMRT。A small randomized controlled trial had reported significantly lower normal organ dose when IMRT was compared to 3DCRT [https://pubmed.ncbi.nlm.nih.gov/21764711/ ]. Furthermore, IMRT was the sole treatment modality in a recent landmark study from China [Int J Radiat Oncol Biol Phys. 2023 Apr 1;115(5):1129-1137] and was advocated in another recent landmark study from Dutch [J Clin Oncol . 2021 Sep 1;39(25):2816-2824]. IMRT was also preferred [over 3DCRT] by the European guideline [ref-2 of the current manuscript] according to its statement “RT should be delivered using 3D conformal RT, but intensity modulated RT or volumetric arc therapy are preferred to better minimise the radiation dose to critical normal tissues.”. The American guideline [NCCN Esophageal cancer 2024v1] also stated “IMRT is now standardly used in the preoperative, definitive, and postoperative treatment of esophageal and esophagogastric cancer”.

The reviewer is not a Japanese and is not sure if 3DCRT (rather than IMRT) was really the standard of radiotherapy modality in Japan, but even so, the results of this study [comparing proton vs 3DCRT] will not be generalizable to many other countries where IMRT was the mainstream of radiotherapy modality for esophageal cancer.

→Certainly, there are many reports on the treatment of esophageal cancer using IMRT. However, as cited from [8], 3DCRT is still often used in Japan for esophageal cancer, especially thoracic esophageal cancer, due to the risk of radiation pneumonitis, and another study, as described in the background in [J Radiat Res. 2022 Jul; 63(4): 646-656.], also shows that 3DCRT is the standard and the application of IMRT to thoracic esophageal cancer is still in the research stage. In addition, we would like to add this article to the references and add to the manuscript that it is still in the research phase.

In addition, the detailed Japanese version of the Japanese guidelines for esophageal cancer, which is the same as [1], the question “Should chemoradiotherapy using PBT be considered for patients with poor cardiopulmonary function? (CQ32)", it states, ”It is weakly recommended that chemoradiotherapy using PBT should be considered for patients with thoracoabdominal esophageal cancer who have poor cardiopulmonary function. ”

#2 “Adverse Events ([CTCAE] ver. 4.0) adverse events of RP, PE, and PCE were reported to be 9%, 16%, and 4%, respectively, for 3D-CRT [3] and 0.5%, 0%, and 1%, respectively, for PBT [5].” >> Adverse Events (CTCAE ver. 4.0) of RP, PE, and PCE were reported to be 9%, 16%, and 4%, respectively, for 3D-CRT [3] and 0.5%, 0%, and 1%, respectively, for PBT [5].? Furthermore, please justify and clarify. Please note CTCv2 was used in ref-3 & CTC v5 was in use currently [https://ctep.cancer.gov/protocoldevelopment/electronic_applications/ctc.htm#ctc_60 accessed 2024/3/22]

→We thank you for your important point and your point is correct. However, we did not see any difference in the evaluation of radiation pneumonitis, pericardial effusion, and pleural effusion under the two adverse event evaluation criteria, so we did not consider it problematic. We have also noted this in the Manuscript.

#3 “Every patient with or without adverse events was moved to best supportive care (BSC), followed by death” >> This was not compatible with the Japanese guideline published in 2023 [Esophagus. 2023 Jul;20(3):343-372] Fig 2 in which salvage therapy was used for residual/recurrence.

→The modeling assumption in this study is that there is no difference in OS and PFS between 3DCRT and PBT, and that recurrence and secondary treatment do not differ between the two groups, are “cancelled out,” and need not be considered. This point is stated as a limitation in “4. discussion”. Our original statement may be unclear, and we clarified the text.

#4 “the clinical evidence supporting the reduction in adverse events with PBT was based on retrospective studies” >> suggest to comment on randomized trials such as J Clin Oncol. 2020 May 10;38(14):1569-1579, although vs IMRT and out of Japan

→ As you pointed out, the clinical evidence for PBT is based on retrospective studies. And, we thank you for the information you provided. However, as you stated, the data is from outside Japan and the irradiation doses are different between Western countries and East Asia, including Japan, so we considered that we could not adopt the data.

[J Radiat Res. 2023 Jun 16;64(Supplement_1):i16-i24.], although the data on PBT are also included in the data on the treatment of carbon ion therapy and combined X-ray therapy, a prospective registry analysis was conducted and the frequency of Grade 3 radiation pneumonitis and pericardial effusions is generally similar to the data used in this study. We will add this to the discussion.

#5 Fig 1. What is the content of fig 1b Markov model? Not seen in the manuscript.

→The second and subsequent paragraphs of “2.3 model structure” are the contents of 1b. Please reconfirm.

#6 Suggest to provide some kind of checklist if not yet [such as CHEERS 2022, Pharmacoeconomics. 2022 Jun;40(6):601-609] as a supplement to ensure the readers regarding the quality of the current manuscript

→ We have already submitted it to the editor. We would like to ask you to check it again.

**Reviewer #2**: In this paper, the authors conduct a cost effectiveness study of PBT in Japan, finding that PBT is cost effective for advanced esophagus cancer in the lower cost schema, but not in the higher cost schema. The study is really cleanly done, very well written, and tempered in its conclusions. The authors should be commended for their work. I have a few minor suggestions:
1. Perhaps highlight in the limitations that the OS benefit for PBT was really only based on one study, that is, Ono et al, in Ref 5. This is fine, but should be noted as a limitation, since their findings and the utility of PBT from OS standpoint are hinged on this one study.

→Thanks for your comments. You are correct and we will include this as a limitation. In addition, [J Radiat Res. 2023 Jun 16;64(Supplement_1):i16-i24.], which includes mixed data on PBT in combination with carbon-ion therapy and x-ray therapy, conducted a prospective registry analysis and found that the frequency of Grade 3 radiation pneumonitis and pericardial effusions is generally similar to the data used in this study. We will add this to the discussion.

2. The authors make an important observation in the discussion that cost-effectiveness may be different if 5040cGy were used commonly rather than 6000cGy, given the differences in epidemiology in Japan. This is an insightful point that the authors bring up. I think the authors can take this a step further and add to the discussion some literature review on 1) cost-effectiveness of PBT for esophagus cancer in in neighboring countries, and 2) cost-effectiveness of PBT for other diseases in Japan and in other nearby countries.
Great work!!

→Thank you for your support of the discussion. We also appreciate your suggestions. We could not find any cost-effectiveness studies of PBT for esophageal cancer as far as we searched. In addition, we found three other reports on the cost-effectiveness of PBT in Japan, including those we have reported in the past, and we will mention them in “4. discussion” as well.

**Reviewer #3:** This paper evaluated the cost-effectiveness of Proton beam therapy (PBT) as a replacement for conventional three-dimensional conformal radiotherapy (3D-CRT) for locally advanced esophageal cancer (LAEC) that is not covered by social insurance, and authors demonstrated that the inclusion of PBT for LAEC in the benefit package of social insurance is cost-effective if a lower fee is applied. Finally, authors concluded that PBT is a cost-effective alternative to 3D-CRT for LAEC and making it available to patients under social insurance could be justifiable. This study dealt with important issue regarding the cost-effectiveness of PBT. However, the design of this study seemed to evaluate the threshold at which PBT is more cost-effective than 3D-CRT for LAEC. The cost calculations should include the expenses for PBT equipment, its reimbursement, and maintenance costs, which are not considered in this study. Therefore, I believe that the conclusions stated by the authors cannot be derived from this study design.

1) Authors assumed two base cases depending on the two existing levels of fees for PBT in social insurance: 2,735,000 Japanese yen (US$20,652) or 1,600,000 yen (US$13,913). However, PBT of LAEC has been not yet covered by national insurance and is still undergoing under the Advanced Medical Care. This cost setting does not reflect the actual costs of PBT. Why did authors assume these cost setting in evaluating the cost-effectiveness?
2) It is easily speculate that running cost of PBT would be higher than 3D-CRT, indicating that this will affect the cost-effectiveness between PBT and 3D-CRT.

→There are PBT facilities across Japan and this paper analyze PBT administered on existing health care infrastructure from payer’s perspective. In our costing, we did not include capital costs such as construction of PBT equipment in the same way as usual cost-effectiveness analysts of hospital care ignore the construction cost of any hospital. We think that choosing this scope of costing is justifiable because it fits to social insurance policy making context as well.

6. PLOS authors have the option to publish the peer review history of their article ([what does this mean?](https://journals.plos.org/plosone/s/editorial-and-peer-review-process#loc-peer-review-history)). If published, this will include your full peer review and any attached files.


If you choose “no”, your identity will remain anonymous but your review may still be made public.


**Do you want your identity to be public for this peer review?** For information about this choice, including consent withdrawal, please see our [Privacy Policy](https://www.plos.org/privacy-policy).

Reviewer #1: No

Reviewer #2: No

Reviewer #3: No
