# Supplementary material for: Cost-utility analysis of proton beam therapy for locally advanced esophageal cancer in Japan
Source: PLoS One. 2024 Sep 27;19(9):e0308961. doi: 10.1371/journal.pone.0308961 (PMC11433116; doi:10.1371/journal.pone.0308961)
Supplement: Attachment — Submitted filename: Response to Reviewers.docx [file pone.0308961.s002.docx]

Dear Dr. Sina Azadnajafabad

Thank you very much for reviewing our research paper. We are pleased to respond to the comments received from the editor and reviewers as follows.

We also have some corrections to report.

We would appreciate it if you could review the paper again.

Kind regards,

Masahide Kondo

From the Author to the Editor

We are pleased to report that we have made some corrections to this paper apart from your previous comments.

These were due to simple misquotations from the literature and research results.

1. In "2.4 Transition Probability," the figures for Transition probability

2. the Probability (rare cancer base-case) in "3. Results.

3. Costs in "Table 1" (we confirmed that other items, such as incremental cost and ICER, were unchanged)

Apologies for any corrections made during the process. Please contact us if you have any questions.

Journal Requirements:

Please review your reference list to ensure that it is complete and correct. If you have cited papers that have been retracted, please include the rationale for doing so in the manuscript text, or remove these references and replace them with relevant current references. Any changes to the reference list should be mentioned in the rebuttal letter that accompanies your revised manuscript. If you need to cite a retracted article, indicate the article’s retracted status in the References list and also include a citation and full reference for the retraction notice.

→We rechecked our references and added one additional reference by revising. We also rechecked the references and their numbers.If you have any doubts, please let us know.

[Note: HTML markup is below. Please do not edit.]

Reviewers' comments:
Reviewer's Responses to Questions

**Comments to the Author**

1. If the authors have adequately addressed your comments raised in a previous round of review and you feel that this manuscript is now acceptable for publication, you may indicate that here to bypass the “Comments to the Author” section, enter your conflict of interest statement in the “Confidential to Editor” section, and submit your "Accept" recommendation.

Reviewer #1: (No Response)

Reviewer #2: All comments have been addressed

2. Is the manuscript technically sound, and do the data support the conclusions?

The manuscript must describe a technically sound piece of scientific research with data that supports the conclusions. Experiments must have been conducted rigorously, with appropriate controls, replication, and sample sizes. The conclusions must be drawn appropriately based on the data presented.

Reviewer #1: Yes

Reviewer #2: Yes

3. Has the statistical analysis been performed appropriately and rigorously?

Reviewer #1: Yes

Reviewer #2: Yes

4. Have the authors made all data underlying the findings in their manuscript fully available?

The [PLOS Data policy](http://www.plosone.org/static/policies.action#sharing) requires authors to make all data underlying the findings described in their manuscript fully available without restriction, with rare exception (please refer to the Data Availability Statement in the manuscript PDF file). The data should be provided as part of the manuscript or its supporting information, or deposited to a public repository. For example, in addition to summary statistics, the data points behind means, medians and variance measures should be available. If there are restrictions on publicly sharing data—e.g. participant privacy or use of data from a third party—those must be specified.

Reviewer #1: Yes

Reviewer #2: Yes

5. Is the manuscript presented in an intelligible fashion and written in standard English?

PLOS ONE does not copyedit accepted manuscripts, so the language in submitted articles must be clear, correct, and unambiguous. Any typographical or grammatical errors should be corrected at revision, so please note any specific errors here.

Reviewer #1: Yes

Reviewer #2: Yes

6. Review Comments to the Author

Please use the space provided to explain your answers to the questions above. You may also include additional comments for the author, including concerns about dual publication, research ethics, or publication ethics. (Please upload your review as an attachment if it exceeds 20,000 characters)

**Reviewer #1:** #1. Generalizability issue [CHEERS 2022 item 26]

Regarding my previous comment-1, the authors had provided references [such as J Radiat Res. 2022 Jul; 63(4): 646-656] to demonstrate that 3DCRT (rather than IMRT) was still the standard radiotherapy modality in Japan.

The reviewer agreed with the authors that there was a lack of high level evidence comparing IMRT vs 3DCRT, although a small randomized trial had reported (>= grade III radiation pneumonitis) was only seen in 3DCRT arm but not IMRT arm [https://pubmed.ncbi.nlm.nih.gov/21764711/]. Therefore, the reviewer agreed that (IMRT vs 3DCRT) was still a subject of research as mentioned by the reference [such as the above J Radiat Res. 2022 Jul; 63(4): 646-656] provided by the authors.

However, the reviewer would also like to point out the IMRT was already the main treatment modality in many countries other than Japan [references see my previous comment-1]. Therefore, the results of the current manuscript [comparing proton vs 3DCRT] will not be generalizable to these countries.

→We appreciate your comments, and we have considered again your point that IMRT is a major treatment modality in many countries. For example, the most recent American society for radiation oncology guideline recommends " In cases where 3-dimensional techniques can not sufficiently reduce the dose to organs at risk to meet required dose objectives, IMRT is recommended."[ <https://doi.org/10.1016/j.prro.2023.10.001> ] and we think it is true that IMRT has spread more rapidly in the United States and other countries than in Japan.

However, as we have mentioned in the past, 3DCRT is still the main treatment method in Japan, so we have added our discussion on this point using Japanese data. The prevalence of IMRT in Japan is still in the 10% range.

As a supplementary note, IMRT has been banned in ongoing clinical trials by Japan clinical oncology group to determine the radiation dose and the extent of radiation for esophageal cancer radiotherapy.

However, we have mentioned that IMRT may need to be considered depending on future changes.

#2 The reviewer assumed fig 1-3 were the same as in version-1 because fig 1-3 were not seen in version-2.

**Reviewer #2:** Thank you for addressing my suggestions. The paper is in excellent shape and is an important contribution to the literature.

7. PLOS authors have the option to publish the peer review history of their article ([what does this mean?](https://journals.plos.org/plosone/s/editorial-and-peer-review-process#loc-peer-review-history)). If published, this will include your full peer review and any attached files.


If you choose “no”, your identity will remain anonymous but your review may still be made public.


**Do you want your identity to be public for this peer review?** For information about this choice, including consent withdrawal, please see our [Privacy Policy](https://www.plos.org/privacy-policy).

Reviewer #1: No

Reviewer #2: No
